# Trilithium thio­arsenate octa­hydrate

**DOI:** 10.1107/S1600536813010921

**Published:** 2013-04-27

**Authors:** Kurt Mereiter

**Affiliations:** a Institute of Chemical Technology and Analytics, Vienna University of Technology, Getreidemarkt 9/164SC, A-1060 Vienna, Austria

## Abstract

The title compound, Li_3_AsS_4_·8H_2_O, is built up from infinite cationic [Li_3_(H_2_O)_8_]^3+^ chains which extend along [001] and are cross-linked by isolated tetra­hedral AsS_4_^3−^ anions *via* O—H⋯S hydrogen bonds. Two Li and two As atoms lie on special positions with site symmetries -1 (1 × Li) and 2 (1 × Li and 2 × As). The [Li_3_(H_2_O)_8_]^3+^ chain contains four independent Li atoms of which two are in octa­hedral and two in tetra­hedral coordination by water O atoms. An outstanding feature of this chain is a linear group of three edge-sharing LiO_6_ octa­hedra to both ends of which two LiO_4_ tetra­hedra are attached by face-sharing. Such groups of composition Li_5_O_16_ are linked into branched chains by means of a further LiO_4_ tetra­hedron sharing vertices with four adjacent LiO_6_ octa­hedra. The Li—O bonds range from 1.876 (5) to 2.054 (6) Å for the LiO_4_ tetra­hedra and from 2.026 (5) to 2.319 (5) Å for the LiO_6_ octa­hedra. The two independent AsS_4_^3−^ anions have As—S bond lengths ranging from 2.1482 (6) to 2.1677 (6) Å [<As—S> = 2.161 (10) Å]. The eight independent water mol­ecules of the structure donate 16 relatively straight O—H⋯S hydrogen bonds to all S atoms of the AsS_4_ tetra­hedra [<O⋯S> = 3.295 (92) Å]. Seven water mol­ecules are in distorted tetra­hedral coordination by two Li and two S; one water mol­ecule has a flat pyramidal coordination by one Li and two S. At variance with related compounds like Schlippe’s salt, Na_3_SbS_4_·9H_2_O, there are neither alkali–sulfur bonds nor O—H⋯O hydrogen bonds in the structure.

## Related literature

For crystal structures of related chalcogenosalt hydrates based on isolated tetra­hedral *XY*_4_ anions, see: Mereiter *et al.* (1979 ▶, 1982 ▶, 1983 ▶); Krebs *et al.* (1990 ▶); Krebs & Jacobsen (1976 ▶); Krebs & Huerter (1980 ▶); Schiwy *et al.* (1973 ▶); Melullis & Dehnen (2007 ▶), Ruzin *et al.* (2006 ▶, 2008 ▶). For the prototype compound Schlippe’s Salt, Na_3_SbS_4_·9H_2_O, see: Schlippe (1821 ▶). For the synthesis of the title compound and early crystallographic data, see: Rémy & Bachet (1968 ▶). For the synthesis and crystal structure of Ba_3_(AsS_4_)_2_·7H_2_O as a precursor of the title compound, see: Mereiter & Preisinger (1992 ▶). For the crystal structure of Na_2_S·9H_2_O, see: Preisinger *et al.* (1982 ▶). For a review on O—H⋯S hydrogen bonds in salt hydrates, see: Mikenda *et al.* (1989 ▶).

## Experimental

### 

#### Crystal data

Li_3_AsS_4_·8H_2_O
*M* *_r_* = 368.11
Monoclinic, 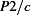

*a* = 10.036 (2) Å
*b* = 10.064 (2) Å
*c* = 14.264 (3) Å
β = 107.30 (1)°
*V* = 1375.5 (5) Å^3^
*Z* = 4
Mo *K*α radiation
μ = 3.09 mm^−1^
*T* = 297 K
0.30 × 0.27 × 0.25 mm

#### Data collection

Philips PW1100 four-circle diffractometer
Absorption correction: for a sphere μR = 0.45 *T*_min_ = 0.51, *T*_max_ = 0.54
5729 measured reflections
4001 independent reflections
2913 reflections with *I* > 2σ(*I*)
*R*_int_ = 0.026
3 standard reflections every 60 min intensity decay: 2.0%

#### Refinement

*R*[*F* ^2^ > 2σ(*F* ^2^)] = 0.028
*wR*(*F* ^2^) = 0.057
*S* = 1.05
4001 reflections
181 parameters
H-atom parameters constrained
Δρ_max_ = 0.61 e Å^−3^
Δρ_min_ = −0.36 e Å^−3^

### 

Data collection: *Philips PW1100 software* (Hornstra & Vossers, 1973 ▶); cell refinement: *LLSQ* (Mereiter, 1992 ▶); data reduction: *PWD* (Mereiter, 1992 ▶); program(s) used to solve structure: *SHELXS97* (Sheldrick, 2008 ▶); program(s) used to refine structure: *SHELXL97* (Sheldrick, 2008 ▶); molecular graphics: *Mercury* (Macrae *et al.*, 2006 ▶) and *DIAMOND* (Brandenburg, 2012 ▶); software used to prepare material for publication: *SHELXL97* and *publCIF* (Westrip, 2010 ▶).

## Supplementary Material

Crystal structure: contains datablock(s) I, global. DOI: 10.1107/S1600536813010921/pj2002sup1.cif

Structure factors: contains datablock(s) I. DOI: 10.1107/S1600536813010921/pj2002Isup2.hkl

Supplementary material file. DOI: 10.1107/S1600536813010921/pj2002Isup3.cml

Additional supplementary materials:  crystallographic information; 3D view; checkCIF report

## Figures and Tables

**Table 1:** Selected bond lengths (Å)

Li1—O2	2.130 (2)
Li1—O1	2.203 (2)
Li1—O3	2.274 (2)
Li2—O3	2.026 (4)
Li2—O2	2.127 (5)
Li2—O5	2.202 (4)
Li2—O4	2.225 (5)
Li2—O6	2.271 (5)
Li2—O7	2.319 (5)
Li3—O4	1.991 (3)
Li3—O1	2.009 (4)
Li4—O8	1.876 (5)
Li4—O5	1.957 (5)
Li4—O6	1.990 (6)
Li4—O7	2.054 (6)
As1—S2	2.1482 (6)
As1—S1	2.1711 (6)
As2—S4	2.1574 (6)
As2—S3	2.1677 (6)

**Table 2:** Hydrogen-bond geometry (Å, °)

*D*—H⋯*A*	*D*—H	H⋯*A*	*D*⋯*A*	*D*—H⋯*A*
O1—H1*A*⋯S1^i^	0.80	2.49	3.262 (2)	163
O1—H1*B*⋯S4^ii^	0.80	2.51	3.287 (2)	163
O2—H2*A*⋯S1^iii^	0.80	2.76	3.531 (2)	162
O2—H2*B*⋯S2	0.80	2.37	3.167 (2)	173
O3—H3*A*⋯S3^iv^	0.80	2.61	3.400 (2)	168
O3—H3*B*⋯S4	0.80	2.39	3.190 (2)	173
O4—H4*A*⋯S1^iii^	0.80	2.51	3.240 (2)	153
O4—H4*B*⋯S3^v^	0.80	2.42	3.207 (2)	170
O5—H5*A*⋯S1	0.80	2.45	3.247 (2)	172
O5—H5*B*⋯S2^iii^	0.80	2.50	3.303 (2)	177
O6—H6*A*⋯S3	0.80	2.57	3.354 (2)	168
O6—H6*B*⋯S4^iv^	0.80	2.54	3.307 (2)	162
O7—H7*A*⋯S1^vi^	0.80	2.49	3.244 (2)	157
O7—H7*B*⋯S3	0.80	2.58	3.333 (2)	157
O8—H8*A*⋯S2^vii^	0.80	2.50	3.253 (2)	157
O8—H8*B*⋯S3^vi^	0.80	2.61	3.394 (2)	166

Symmetry codes: (i) 
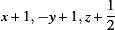
; (ii) 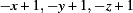; (iii) 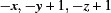; (iv) 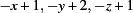; (v) 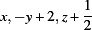; (vi) 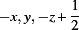; (vii) 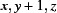.

**Fig. 1.**
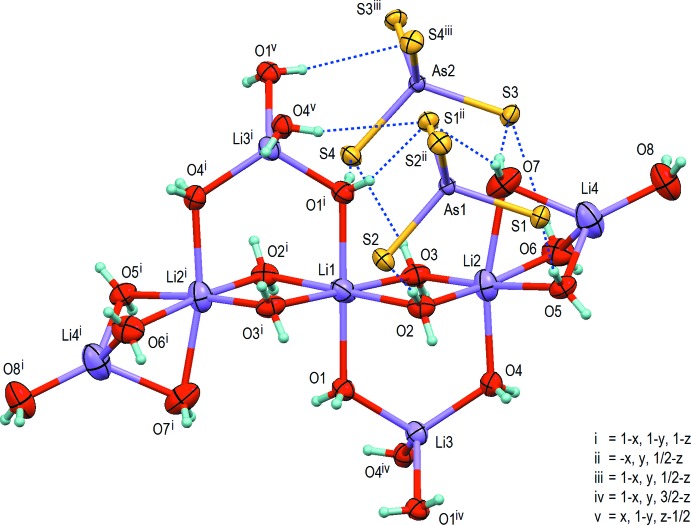
A respresentative part of the structure of Li3AsS4.8H2O with displacement ellipsoids for the non-hydrogen atoms drawn at the 50% probability level. Symmetry codes are given on the lower right. Hydrogen bonds in this assembly are shown as dashed lines.

**Fig. 2.**
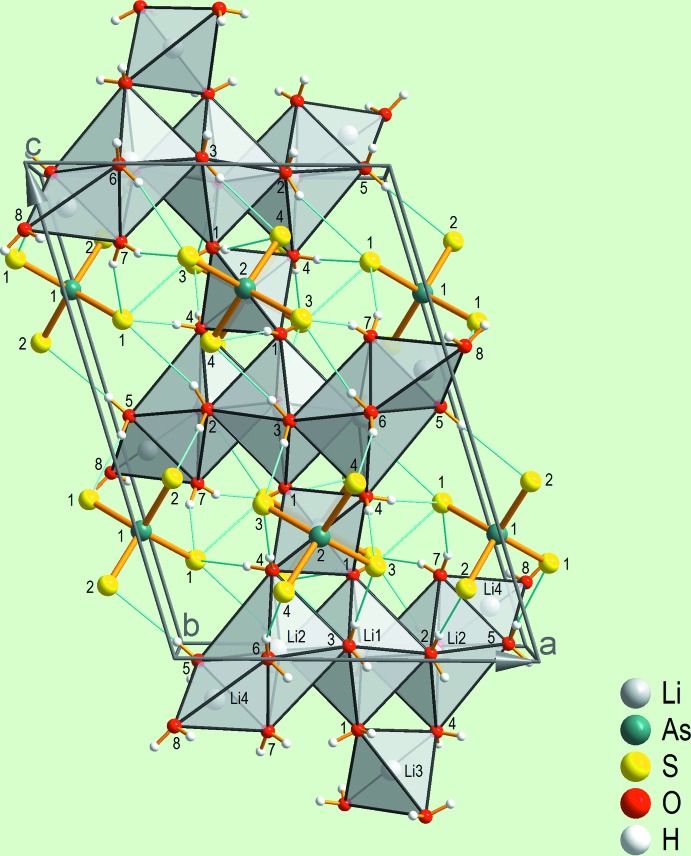
A packing diagram of Li3AsS4.8H2O viewed along the b-axis showing also the hydrogen bonds. Only the label numbers are given for As, S, and O atoms.

**Fig. 3.**
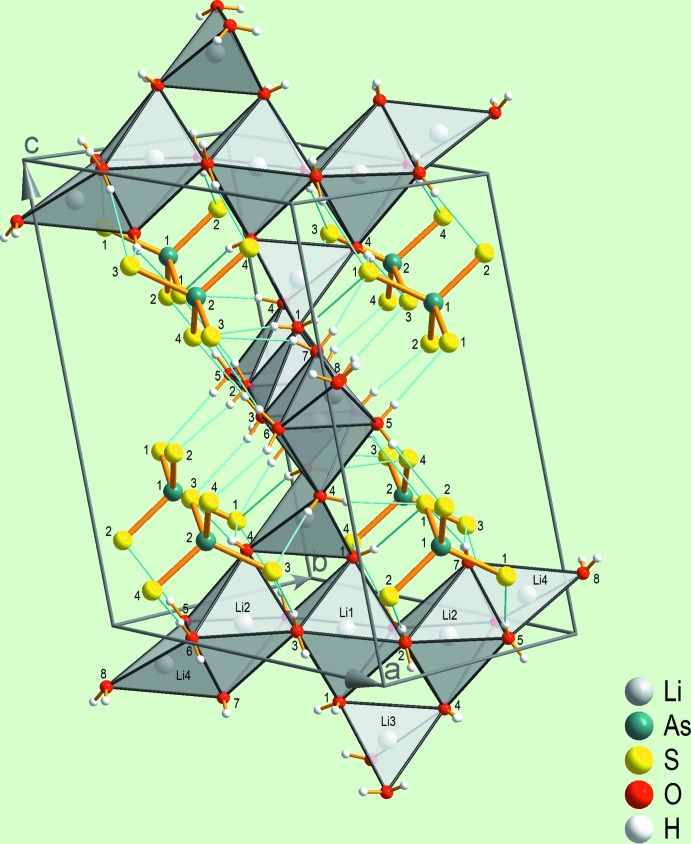
A packing diagram of Li3AsS4.8H2O viewed approximately along to [110] showing also the hydrogen bonds. Only the label numbers are given for As, S, and O atoms.

**Fig. 4.**
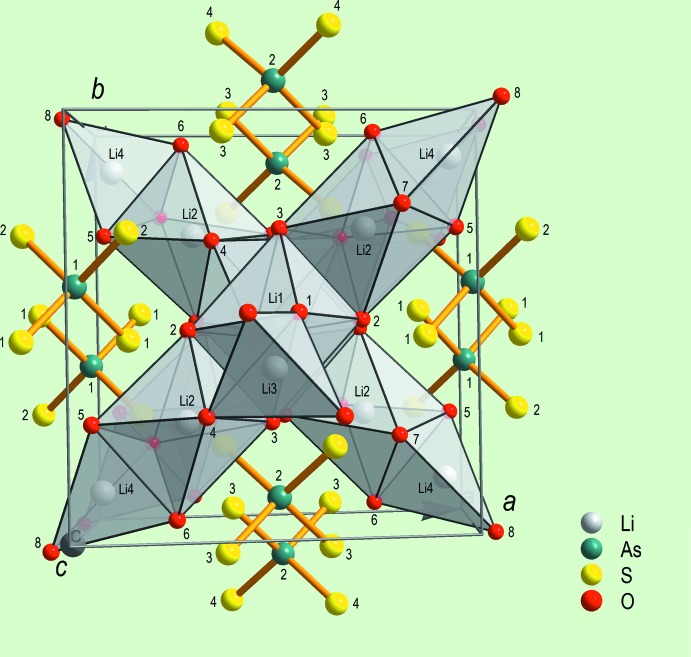
View of the structure of Li3AsS4.8H2O along [001]. H-atoms omitted for clarity. Li3, As1 and As2 lie on twofold axes parallel [010], and Li1 on 1 at x,y,z = 1/2,1/2,1/2.

## supplementary crystallographic information

### Comment

The title compound, Li_3_AsS_4_.8H_2_O, belongs to a group of alkali chalcogenosalt hydrates with tetrahedral XY_4_ anions, *X* = P(V), As(V), Sb(V), Ge(IV), Sn(IV) and Y = S^2-^, Se^2-^, Te^2-^, of which Schlippe's salt, Na_3_SbS_4_.9H_2_O (Schlippe, 1821; Mereiter *et al.*, 1979) can be considered as the prototype. Other representatives with known crystal structures are Na_3_PS_4_.8H_2_O (Mereiter *et al.*, 1983), Na_3_AsS_4_.8H_2_O (Mereiter *et al.*, 1982), Na_3_AsSe_4_.9H_2_O (Krebs *et al.*, 1990), Na_4_SnS_4_.14H_2_O (Schiwy *et al.*, 1973), Na_4_GeSe_4_.14H_2_O (Krebs & Jacobsen, 1976), Na_4_SnSe_4_.16H_2_O (Krebs & Huerter, 1980), K_4_GeSe_4_.4H_2_O (Melullis & Dehnen, 2007), Rb_4_SnTe_4_.2H_2_O (Ruzin *et al.*, 2006), K_4_SnS_4_.4H_2_O (Ruzin *et al.*, 2008), to mention only some examples.

In context with previous work (Mereiter & Preisinger, 1992) the title compound Li_3_AsS_4_.8H_2_O was prepared and its crystal structure was determined. An early report on the synthesis and crystal data of this compound was given by Rémy & Bachet (1968).

Li_3_AsS_4_.8H_2_O crystallizes in the infrequent centrosymmetric monoclinic space group *P*2/c (No. 13). The crystal structure contains four independent Li atoms, two independent AsS_4_ tetrahedra and eight independent water molecules in the asymmetric unit. Li1 lies on a centre of inversion while Li3, As1, and As2 are located on twofold axes. All other constituents comprising Li2 and Li4, four S, and eight H_2_O are in general position. A view of a characteristic part of the crystal structure containing all constituents is shown in Fig. 1. All lithium atoms are exclusively coordinated by the oxygen atoms of the water molecules but not by any sulfur atom. Li1 and Li2 form Li(H_2_O)_6_ octahedra while Li3 and Li4 form Li(H_2_O)_4_ tetrahedra. The Li1(H_2_O)_6_ octahedron shares edges with two adjacent Li2(H_2_O)_6_ octahedra, and they in turn share faces with two Li4(H_2_O)_4_ tetrahedra. This gives rise to characteristic polyhedral pentamers Li_5_(H_2_O)_16_ which are linked by the Li3(H_2_O)_4_ tetrahedra *via* vertex sharing with four adjacent Li(H_2_O)_6_ octahedra to form infinite branched chains of the composition Li_6_(H_2_O)_16_ or Li_3_(H_2_O)_8_ (Figs. 2 and 3). The chains extend along [001] and have the Li_5_(H_2_O)_16_ fragments oriented in a criss-cross fashion (Figs. 3 and 4). Embedded between the chains are the tetrahedral AsS_4_^3-^ anions, which are are anchored exclusively by O—H···S type hydrogen bonds donated by the water molecules. The sulfur atoms receive either three (S2, S4) or five (S1, S3) hydrogen bonds so that each AsS_4_^3-^ anion receives 16 hydrogen bonds of which 8 are symmetry redundant. The eight different water molecules of the structure have mainly distorted tetrahedral coordination environments by two Li cations and two S atoms as hydrogen bond acceptors. Only the water molecule H_2_O8 differs from this behaviour by being bonded to only one Li and two S atoms within a distorted trigonal pyramidal coordination.

The Li—O bonds range from 2.026 (5) to 2.319 (5) Å for the LiO_6_ octahedra and from 1.876 (5) to 2.054 (6) Å for the LiO_4_ tetrahedra (Table 1). The mean Li—O bond lengths are 2.202 (65), 2.195 (105), 2.000 (10) and 1.969 (74) Å for Li1 through Li4. The coordination figures of Li1 and Li3 are relatively regular (O—Li—O bond angles for Li1O_6_ 81.84 (6)–98.16 (6)° and 180°, for Li3O_4_ 101.7 (2)–114.3 (3)°) while those of Li2 and Li4 are notably distorted due to the face-sharing link between them (O—Li—O bond angles for Li2O_6_ 72.7 (2)–100.6 (2)° and 167.3 (2)–174.8 (3)°, for Li4O_4_ 84.6 (2)–129.8 (2)°; Li2···Li4 = 2.698 (7) Å). The two independent AsS_4_^3-^ anions have As—S bond distances from 2.1482 (6) to 2.1677 (6) Å, <As—S> = 2.161 (10) Å. The two shorter As—S bonds in each tetrahedron are to sulfur atoms receiving three hydrogen bonds (S2, S4) while the longer As—S bonds are to sulfur atoms receiving five hydrogen bonds (S1, S3). The As—S bond lengths and the S—As—S bond angles (106.72 (3)–111.42 (2) and 106.15 (2)–112.69 (2)° for As1 and As2, respectively) show that the two tetrahedra are relatively regular. They agree in their dimensions with related thioarsenates (Mereiter *et al.*, 1982; Mereiter & Preisinger, 1992). The eight independent water molecules of the structure donate 16 relatively straight O—H···S hydrogen bonds to all S atoms of the AsS_4_ tetrahedra, O···S = 3.167 (2) – 3.531 (2) Å, <O···S> = 3.295 (92) Å and O—H···S = 153–177° (Table 2; Figs. 2 and 3). These dimensions fit well into the pattern of O—H···S hydrogen bonds in sulfosalt hydrates reported by Mikenda *et al.* (1989).

The structure of title compound Li_3_AsS_4_.8H_2_O adds a new facet to the very diverse structural chemistry of the chalcogenosalt hydrates with tetrahedral XY_4_ anions defined above. The two isochemical relatives of the title compound, Na_3_PS_4_.8H_2_O (Mereiter *et al.*, 1983) and Na_3_AsS_4_.8H_2_O (Mereiter *et al.*, 1982), represent an isostructural pair built up from NaS_2_(H_2_O)_4_, NaS(H_2_O)_5_ and Na(H_2_O)_6_ octahedra which form together with the PS_4_/AsS_4_ tetrahedra undulating layers. The PS_4_/AsS_4_ tetrahedron is linked with three S atoms to the Na cations. The water molecules are mostly bonded to two Na and donate pairs of O—H···S hydrogen bonds. One water molecule is bonded to only one Na and accepts in compensation an O—H···O hydrogen bond. Schlippe's salt, Na_3_SbS_4_.9H_2_O (Mereiter *et al.*, 1979) and the isostructural selenoarsenate (Krebs *et al.*, 1990) contain per formula unit one water more than the title compound. They are built up from tripledeckers of facesharing octahedra - namely a Na(H_2_O)_6_ octahedron which shares two opposite faces with a Na(H_2_O)_6_ and a NaS_3_(H_2_O)_3_ octahedron. The tetrahedral SbS_4_ anion is bonded with three S atoms to three tripledeckers and links them into a complicated framework of cubic symmetry. Here again only one S atom is not cation-bonded and the structure contains independent five O—H···S and one O—H···O bonds. The compounds Na_4_SnS_4_.14H_2_O (Schiwy *et al.*, 1973), Na_4_GeSe_4_.14H_2_O (Krebs & Jacobsen, 1976), and Na_4_SnSe_4_.16H_2_O (Krebs & Huerter, 1980) can be seen as variants of the Na-water triple deckers in Schlippe's salt and only in Na_4_SnSe_4_.16H_2_O these triple deckers form Na—H_2_O polymers without any Na-chalcogen bonds. Alkali sulfosalts with cations larger than Na – *e.g.* K_4_GeSe_4_.4H_2_O (Melullis & Dehnen, 2007), Rb_4_SnTe_4_.2H_2_O (Ruzin *et al.*, 2006), or K_4_SnS_4_.4H_2_O (Ruzin *et al.*, 2008) – contain less water per cation and adopt alkali-water structures with higher coordination numbers than 6 for the cation sites and they involve therefore increasingly alkali-chalcogen bonds. Although not a sulfo-salt, Na_2_S.9H_2_O may be mentioned here for comparison with Li_3_AsS_4_.8H_2_O because it is built up from octahedral chains Na(H_2_O)_5_ (corner-sharing spiral chains) and Na(H_2_O)_4_ (edge-sharing spiral chains) held together by isolated sulfide ions *via* many O—H···S bonds and by some O—H···O bonds (Preisinger *et al.*, 1982).

### Experimental

The title compound was prepared by mixing dilute aqueous solutions of Ba_3_(AsS_4_)_2_.7H_2_O (Mereiter & Preisinger, 1992) and Li_2_SO_4_.H_2_O in stoichiometric amounts. After removing BaSO_4_ by filtration, the solution was preconcentrated in a rotavapor under reduced pressure and then filtered. Crystallization by room temperature evaporation in an exsiccator over conc. H_2_SO_4_ as desiccant gave the desired Li_3_AsS_4_.8H_2_O in the form of colourless prisms. For structure analysis, a fragment was rounded to an oval by turning it on wet filter paper. Another preparation method for Li_3_AsS_4_.8H_2_O was reported by Rémy & Bachet (1968).

### Refinement

All water molecules were idealized to have O—H = 0.80 Å and H—O—H = 105.0° and were then refined as rigid groups using AFIX 6 of *SHELXL97* (Sheldrick, 2008). A common *U*_iso_ for the two H-atoms of each water molecule was used and refined.

### Crystal data

**Table:**

Li_3_AsS_4_·8H_2_O	*F*(000) = 744
*M**_r_* = 368.11	*D*_x_ = 1.778 Mg m^−^^3^
Monoclinic, *P*2/*c*	Mo *K*α radiation, λ = 0.71073 Å
Hall symbol: -P 2yc	Cell parameters from 34 reflections
*a* = 10.036 (2) Å	θ = 9.1–26.2°
*b* = 10.064 (2) Å	µ = 3.09 mm^−^^1^
*c* = 14.264 (3) Å	*T* = 297 K
β = 107.30 (1)°	Oval, colourless
*V* = 1375.5 (5) Å^3^	0.30 × 0.27 × 0.25 mm
*Z* = 4	

### Data collection

**Table:**

Philips PW1100 four-circle diffractometer	2913 reflections with *I* > 2σ(*I*)
Radiation source: fine-focus sealed tube	*R*_int_ = 0.026
Graphite monochromator	θ_max_ = 30.0°, θ_min_ = 2.0°
ω–2θ scans	*h* = −14→13
Absorption correction: for a sphere µR = 0.45	*k* = −14→14
*T*_min_ = 0.51, *T*_max_ = 0.54	*l* = 0→20
5729 measured reflections	3 standard reflections every 60 min
4001 independent reflections	intensity decay: 2.0%

### Refinement

**Table:**

Refinement on *F*^2^	Secondary atom site location: difference Fourier map
Least-squares matrix: full	Hydrogen site location: inferred from neighbouring sites
*R*[*F*^2^ > 2σ(*F*^2^)] = 0.028	H-atom parameters constrained
*wR*(*F*^2^) = 0.057	*w* = 1/[σ^2^(*F*_o_^2^) + (0.0179*P*)^2^ + 0.5909*P*] where *P* = (*F*_o_^2^ + 2*F*_c_^2^)/3
*S* = 1.05	(Δ/σ)_max_ = 0.001
4001 reflections	Δρ_max_ = 0.61 e Å^−^^3^
181 parameters	Δρ_min_ = −0.36 e Å^−^^3^
0 restraints	Extinction correction: *SHELXL97* (Sheldrick, 2008), Fc^*^=kFc[1+0.001xFc^2^λ^3^/sin(2θ)]^-1/4^
Primary atom site location: structure-invariant direct methods	Extinction coefficient: 0.0081 (3)

### Special details

**Table:**

Geometry. All e.s.d.'s (except the e.s.d. in the dihedral angle between two l.s. planes) are estimated using the full covariance matrix. The cell e.s.d.'s are taken into account individually in the estimation of e.s.d.'s in distances, angles and torsion angles; correlations between e.s.d.'s in cell parameters are only used when they are defined by crystal symmetry. An approximate (isotropic) treatment of cell e.s.d.'s is used for estimating e.s.d.'s involving l.s. planes.
Refinement. Refinement of *F*^2^ against ALL reflections. The weighted *R*-factor *wR* and goodness of fit *S* are based on *F*^2^, conventional *R*-factors *R* are based on *F*, with *F* set to zero for negative *F*^2^. The threshold expression of *F*^2^ > σ(*F*^2^) is used only for calculating *R*-factors(gt) *etc*. and is not relevant to the choice of reflections for refinement. *R*-factors based on *F*^2^ are statistically about twice as large as those based on *F*, and *R*- factors based on ALL data will be even larger.

### Fractional atomic coordinates and isotropic or equivalent isotropic displacement parameters (Å^2^)

**Table:**

	*x*	*y*	*z*	*U*_iso_*/*U*_eq_	
Li1	0.5000	0.5000	0.5000	0.0393 (13)	
Li2	0.2821 (4)	0.7213 (4)	0.5102 (4)	0.0443 (10)	
Li3	0.5000	0.5984 (5)	0.7500	0.0311 (11)	
Li4	0.0724 (5)	0.8832 (5)	0.4126 (5)	0.0610 (14)	
As1	0.0000	0.40273 (3)	0.2500	0.01865 (8)	
As2	0.5000	0.91402 (2)	0.2500	0.01887 (8)	
S1	−0.12581 (5)	0.53147 (5)	0.31185 (4)	0.02508 (11)	
S2	0.13565 (5)	0.27910 (5)	0.36066 (4)	0.02849 (12)	
S3	0.37068 (5)	1.03848 (5)	0.31147 (4)	0.02615 (12)	
S4	0.64151 (6)	0.79087 (5)	0.35891 (4)	0.02873 (12)	
O1	0.55849 (15)	0.47247 (15)	0.66032 (12)	0.0297 (3)	
H1A	0.6400	0.4629	0.6874	0.054 (6)*	
H1B	0.5239	0.4023	0.6655	0.054 (6)*	
O2	0.28906 (17)	0.51048 (16)	0.50233 (12)	0.0330 (3)	
H2A	0.2693	0.4923	0.5512	0.068 (8)*	
H2B	0.2494	0.4564	0.4627	0.068 (8)*	
O3	0.48739 (17)	0.72351 (17)	0.51911 (12)	0.0359 (4)	
H3A	0.5233	0.7696	0.5654	0.077 (8)*	
H3B	0.5211	0.7466	0.4775	0.077 (8)*	
O4	0.33730 (16)	0.70514 (14)	0.67279 (12)	0.0303 (3)	
H4A	0.2703	0.6689	0.6804	0.051 (6)*	
H4B	0.3423	0.7745	0.7010	0.051 (6)*	
O5	0.05507 (17)	0.72354 (16)	0.48637 (12)	0.0344 (4)	
H5A	0.0167	0.6700	0.4459	0.059 (7)*	
H5B	0.0110	0.7247	0.5248	0.059 (7)*	
O6	0.2558 (2)	0.9454 (2)	0.49965 (14)	0.0486 (5)	
H6A	0.2939	0.9720	0.4614	0.107 (11)*	
H6B	0.2680	1.0022	0.5406	0.107 (11)*	
O7	0.1906 (2)	0.7739 (2)	0.34519 (16)	0.0535 (5)	
H7A	0.1517	0.7256	0.3009	0.112 (12)*	
H7B	0.2329	0.8251	0.3220	0.112 (12)*	
O8	−0.05671 (19)	1.0198 (2)	0.36351 (18)	0.0573 (6)	
H8A	−0.0190	1.0806	0.3455	0.102 (11)*	
H8B	−0.1288	1.0100	0.3210	0.102 (11)*	

### Atomic displacement parameters (Å^2^)

**Table:**

	*U*^11^	*U*^22^	*U*^33^	*U*^12^	*U*^13^	*U*^23^
Li1	0.035 (3)	0.041 (3)	0.041 (3)	0.003 (3)	0.010 (2)	0.012 (3)
Li2	0.035 (2)	0.038 (2)	0.059 (3)	0.0012 (18)	0.013 (2)	0.001 (2)
Li3	0.029 (3)	0.030 (3)	0.032 (3)	0.000	0.006 (2)	0.000
Li4	0.049 (3)	0.046 (2)	0.086 (4)	0.005 (2)	0.017 (3)	0.018 (3)
As1	0.01756 (13)	0.01845 (13)	0.01937 (15)	0.000	0.00463 (11)	0.000
As2	0.02138 (14)	0.01735 (13)	0.01920 (15)	0.000	0.00807 (11)	0.000
S1	0.0240 (2)	0.0256 (2)	0.0277 (3)	0.00128 (18)	0.0108 (2)	−0.0028 (2)
S2	0.0273 (3)	0.0254 (2)	0.0287 (3)	0.0044 (2)	0.0022 (2)	0.0044 (2)
S3	0.0294 (3)	0.0243 (2)	0.0287 (3)	0.00180 (19)	0.0148 (2)	−0.0032 (2)
S4	0.0294 (3)	0.0266 (2)	0.0286 (3)	0.0047 (2)	0.0063 (2)	0.0071 (2)
O1	0.0293 (8)	0.0294 (7)	0.0276 (8)	0.0006 (6)	0.0042 (6)	0.0006 (6)
O2	0.0358 (8)	0.0319 (8)	0.0293 (8)	−0.0075 (7)	0.0066 (7)	−0.0028 (7)
O3	0.0358 (9)	0.0438 (9)	0.0290 (9)	−0.0110 (7)	0.0110 (7)	−0.0023 (8)
O4	0.0314 (8)	0.0250 (7)	0.0365 (9)	−0.0023 (6)	0.0130 (7)	−0.0017 (6)
O5	0.0385 (9)	0.0360 (8)	0.0311 (9)	−0.0074 (7)	0.0140 (7)	−0.0056 (7)
O6	0.0590 (12)	0.0413 (9)	0.0406 (11)	−0.0078 (9)	0.0076 (10)	−0.0058 (9)
O7	0.0693 (14)	0.0463 (11)	0.0568 (13)	−0.0217 (10)	0.0367 (11)	−0.0157 (10)
O8	0.0377 (10)	0.0424 (11)	0.0836 (16)	0.0008 (9)	0.0052 (10)	0.0137 (11)

### Geometric parameters (Å, º)

**Table:**

Li1—O2	2.130 (2)	As1—S1^iii^	2.1711 (6)
Li1—O2^i^	2.130 (2)	As1—S1	2.1711 (6)
Li1—O1	2.203 (2)	As2—S4^iv^	2.1574 (6)
Li1—O1^i^	2.203 (2)	As2—S4	2.1574 (6)
Li1—O3	2.274 (2)	As2—S3	2.1677 (6)
Li1—O3^i^	2.274 (2)	As2—S3^iv^	2.1677 (6)
Li2—O3	2.026 (4)	O1—H1A	0.80
Li2—O2	2.127 (5)	O1—H1B	0.80
Li2—O5	2.202 (4)	O2—H2A	0.80
Li2—O4	2.225 (5)	O2—H2B	0.80
Li2—O6	2.271 (5)	O3—H3A	0.80
Li2—O7	2.319 (5)	O3—H3B	0.80
Li3—O4^ii^	1.991 (3)	O4—H4A	0.80
Li3—O4	1.991 (3)	O4—H4B	0.80
Li3—O1	2.009 (4)	O5—H5A	0.80
Li3—O1^ii^	2.009 (4)	O5—H5B	0.80
Li4—O8	1.876 (5)	O6—H6A	0.80
Li4—O5	1.957 (5)	O6—H6B	0.80
Li4—O6	1.990 (6)	O7—H7A	0.80
Li4—O7	2.054 (6)	O7—H7B	0.80
As1—S2^iii^	2.1482 (6)	O8—H8A	0.80
As1—S2	2.1482 (6)	O8—H8B	0.80
			
O2—Li1—O2^i^	180.0	S4—As2—S3	112.69 (2)
O2—Li1—O1^i^	92.91 (6)	S4^iv^—As2—S3^iv^	112.69 (2)
O2^i^—Li1—O1^i^	87.09 (6)	S4—As2—S3^iv^	106.15 (2)
O2—Li1—O1	87.09 (6)	S3—As2—S3^iv^	109.40 (3)
O2^i^—Li1—O1	92.91 (6)	Li3—O1—Li1	122.93 (10)
O1^i^—Li1—O1	180.0	Li3—O1—H1A	102.7
O2—Li1—O3^i^	98.16 (6)	Li1—O1—H1A	115.8
O2^i^—Li1—O3^i^	81.84 (6)	Li3—O1—H1B	106.3
O1^i^—Li1—O3^i^	90.41 (6)	Li1—O1—H1B	102.6
O1—Li1—O3^i^	89.59 (6)	H1A—O1—H1B	105.0
O2—Li1—O3	81.84 (6)	Li2—O2—Li1	95.65 (13)
O2^i^—Li1—O3	98.16 (6)	Li2—O2—H2A	99.3
O1^i^—Li1—O3	89.59 (6)	Li1—O2—H2A	120.5
O1—Li1—O3	90.41 (6)	Li2—O2—H2B	134.3
O3^i^—Li1—O3	180.0	Li1—O2—H2B	104.1
O3—Li2—O2	88.03 (18)	H2A—O2—H2B	105.0
O3—Li2—O5	174.8 (3)	Li2—O3—Li1	94.22 (14)
O2—Li2—O5	92.89 (17)	Li2—O3—H3A	105.0
O3—Li2—O4	90.12 (18)	Li1—O3—H3A	130.2
O2—Li2—O4	88.84 (18)	Li2—O3—H3B	127.1
O5—Li2—O4	94.98 (19)	Li1—O3—H3B	98.5
O3—Li2—O6	95.14 (18)	H3A—O3—H3B	105.0
O2—Li2—O6	172.9 (3)	Li3—O4—Li2	121.68 (14)
O5—Li2—O6	83.40 (16)	Li3—O4—H4A	105.3
O4—Li2—O6	97.45 (19)	Li2—O4—H4A	102.5
O3—Li2—O7	98.6 (2)	Li3—O4—H4B	105.9
O2—Li2—O7	100.6 (2)	Li2—O4—H4B	114.8
O5—Li2—O7	76.20 (15)	H4A—O4—H4B	105.0
O4—Li2—O7	167.3 (2)	Li4—O5—Li2	80.7 (2)
O6—Li2—O7	72.74 (15)	Li4—O5—H5A	105.4
O4^ii^—Li3—O4	114.7 (3)	Li2—O5—H5A	111.0
O4^ii^—Li3—O1	110.23 (7)	Li4—O5—H5B	120.8
O4—Li3—O1	109.58 (7)	Li2—O5—H5B	130.7
O4^ii^—Li3—O1^ii^	109.58 (7)	H5A—O5—H5B	105.0
O4—Li3—O1^ii^	110.23 (7)	Li4—O6—Li2	78.26 (19)
O1—Li3—O1^ii^	101.7 (2)	Li4—O6—H6A	102.8
O8—Li4—O5	129.8 (3)	Li2—O6—H6A	107.9
O8—Li4—O6	114.2 (3)	Li4—O6—H6B	126.3
O5—Li4—O6	97.9 (3)	Li2—O6—H6B	132.2
O8—Li4—O7	130.4 (3)	H6A—O6—H6B	105.0
O5—Li4—O7	88.2 (2)	Li4—O7—Li2	75.9 (2)
O6—Li4—O7	84.6 (2)	Li4—O7—H7A	118.8
S2^iii^—As1—S2	109.21 (3)	Li2—O7—H7A	128.0
S2^iii^—As1—S1^iii^	111.42 (2)	Li4—O7—H7B	107.4
S2—As1—S1^iii^	109.04 (2)	Li2—O7—H7B	117.9
S2^iii^—As1—S1	109.04 (2)	H7A—O7—H7B	105.0
S2—As1—S1	111.42 (2)	Li4—O8—H8A	109.9
S1^iii^—As1—S1	106.72 (3)	Li4—O8—H8B	124.1
S4^iv^—As2—S4	109.87 (4)	H8A—O8—H8B	105.0
S4^iv^—As2—S3	106.15 (2)		

Symmetry codes: (i) −*x*+1, −*y*+1, −*z*+1; (ii) −*x*+1, *y*, −*z*+3/2; (iii) −*x*, *y*, −*z*+1/2; (iv) −*x*+1, *y*, −*z*+1/2.

### Hydrogen-bond geometry (Å, º)

**Table:**

*D*—H···*A*	*D*—H	H···*A*	*D*···*A*	*D*—H···*A*
O1—H1*A*···S1^v^	0.80	2.49	3.262 (2)	163
O1—H1*B*···S4^i^	0.80	2.51	3.287 (2)	163
O2—H2*A*···S1^vi^	0.80	2.76	3.531 (2)	162
O2—H2*B*···S2	0.80	2.37	3.167 (2)	173
O3—H3*A*···S3^vii^	0.80	2.61	3.400 (2)	168
O3—H3*B*···S4	0.80	2.39	3.190 (2)	173
O4—H4*A*···S1^vi^	0.80	2.51	3.240 (2)	153
O4—H4*B*···S3^viii^	0.80	2.42	3.207 (2)	170
O5—H5*A*···S1	0.80	2.45	3.247 (2)	172
O5—H5*B*···S2^vi^	0.80	2.50	3.303 (2)	177
O6—H6*A*···S3	0.80	2.57	3.354 (2)	168
O6—H6*B*···S4^vii^	0.80	2.54	3.307 (2)	162
O7—H7*A*···S1^iii^	0.80	2.49	3.244 (2)	157
O7—H7*B*···S3	0.80	2.58	3.333 (2)	157
O8—H8*A*···S2^ix^	0.80	2.50	3.253 (2)	157
O8—H8*B*···S3^iii^	0.80	2.61	3.394 (2)	166

Symmetry codes: (i) −*x*+1, −*y*+1, −*z*+1; (iii) −*x*, *y*, −*z*+1/2; (v) *x*+1, −*y*+1, *z*+1/2; (vi) −*x*, −*y*+1, −*z*+1; (vii) −*x*+1, −*y*+2, −*z*+1; (viii) *x*, −*y*+2, *z*+1/2; (ix) *x*, *y*+1, *z*.
